# Promoting Positive Adolescent Mental Health (PPAMH!) for School Climate: protocol for a feasibility randomised control trial in secondary schools

**DOI:** 10.3310/nihropenres.13678.1

**Published:** 2024-10-24

**Authors:** Ruth Wadman, Olivia Taylor, Kristian Hudson, Dan Lewer, Catherine Hewitt, John Pickavance, Joseph Pryce, John Wright, Simon Gilbody

**Affiliations:** 1 Health Sciences, University of York, York, England, YO10 5DD, UK; 2 Bradford Teaching Hospitals NHS Foundation Trust, Bradford Institute for Health Research, Bradford, England, BD9 6RJ, UK; 3 London School of Hygiene & Tropical Medicine, London, England, WC1E 7HT, UK

**Keywords:** School based interventions, School attendance, Mental health, Secondary schools

## Abstract

**Introduction:**

We describe a feasibility randomised controlled trial to determine if it is possible to implement a full cluster-randomised trial of a multi-component whole-school intervention aiming to improve the school climate. The Promoting Positive Adolescent Mental Health (PPAMH!) intervention will be adapted from an intervention previously trialled in India and Australia that reduced bullying, depression and risk behaviours among students.

**Methods:**

We will randomise six secondary schools in Bradford in a feasibility trial. Three schools will receive the intervention and three will act as the ‘usual care’ control condition. We will collect quantitative and qualitative data on the implementation through activity logs and interviews with staff and well-being mentors. We will determine the feasibility of accessing and using routine education data and self-report mental health data as outcomes in the full trial.

**Results:**

This study will provide information about the acceptability and feasibility of delivering and evaluating the school climate intervention in secondary schools.

**Conclusions:**

This is the first study to deliver a promising whole school climate intervention in the UK. The results of this feasibility trial will inform the decision to embark on a full trial and further intervention adaptations as needed.

**Trial registration:**

ISRCTN registry reference ISRCTN14856201

## Plain English Summary

PPAMH! (‘Promoting Positive Adolescent Mental Health) for School Climate encourages a positive environment to support student wellbeing. A ‘well-being mentor’ will work in each school for half of the week. They will do whole-school activities such as assemblies, peer group activities such as workshops, and one-to-one activities with pupils. The intervention focuses on aiding pupils to have their say on school policies and curriculum by creating a participative school environment.

Interventions using a similar approach have been done in India and Australia. We think that PPAMH! could improve student attendance. The research trial in India found that it reduced bullying and feelings of depression. We will adapt the trial to be suitable for schools in Bradford through workshops with students and staff.

This initial trial will involve six secondary schools in Bradford. Schools will be randomly chosen to either being a control school or to receive the intervention. The main outcome is the successful running of the intervention in the schools, for example looking at how many activities were completed and by how many students. We will not study the effect of the intervention on school absences or other aspects of wellbeing. We will interview school staff members who are involved in providing the intervention in the schools and the well-being mentors. This will tell us about barriers to the intervention, how these could be overcome and changes that schools make to the intervention. We will also see how well we are able to access the data we plan to use in the full trial, which is school absences (from routine educational data) and mental health data such as depression and anxiety symptoms (via student surveys). We will plan to do a full trial if the feasibility trial shows the PPAMH! intervention can be successfully set up in schools.

The prevalence of mental health problems is increasing in young people. In the UK, rates of diagnosis for anxiety, depression, attention disorders, and eating disorders doubled between 2003 and 2018 ^1^, while the COVID-19 pandemic and associated restrictions likely had an additional impact ^2^. The Mental Health of Children and Young People in England 2022 report found that 18% of 7- to 16-year-olds and 22% of 17- to 24-year-olds had a probable mental health disorder ^3^. In addition to the impact of mental health problems on young people and their families, young people’s mental health difficulties are associated with large economic costs and impacts on public services including the NHS ^4^. Increasing demand for, and pressures on, specialist services such as Child and Adolescent Mental Health Services (CAMHS) ^5^, has led to young people facing longer waiting times or having no contact with support services.

Most mental health problems start by adolescence, with half established by age 14 ^6– 7^. Elevated mental health symptoms and diagnoses among adolescents are predictive of later mental health problems and are associated with worse outcomes in multiple areas of daily life in adulthood, including educational achievements and employment patterns ^8– 11^. Children and young people with mental health problems are at risk of higher school absence rates. In England in 2021, 13% of children with a probable mental disorder missed more than 15 days of school in one year, compared with 4% of those without a mental health disorder ^12^.

Schools play a crucial role in supporting wellbeing and mental health, with children spending more time in school than any other formal institution. School-based preventative interventions for mental health include universal approaches (for all students), selective interventions targeted at at-risk groups, and indicative (early) interventions for individuals with mental health symptoms.

Universal social and emotional learning (SEL) interventions, that aim to improve social and emotional knowledge and skills, have been found to reduce symptoms of depression and anxiety at least in the short-term but there is limited evidence of the longer-term effect of SEL ^13^. Evidence also indicates that targeted depression- and anxiety-focused school-based interventions, such as targeted cognitive behavioural therapy (CBT) delivered by external experts, can reduce symptoms in the short- and medium-term ^14, 15^. However, the effectiveness of universal school-based CBT preventative interventions is less apparent, with two trials reporting no improvements in mental health symptoms ^16, 17^.

These large-scale evaluations present a mixed picture for the effectiveness of universal school-based interventions. A UK trial of classroom-based mindfulness (MYRIAD trial) found no evidence of benefit in terms of reducing risk of depression or improving socioemotional-behavioural functioning ^18^ and additional analyses suggested the intervention may harm students at risk of mental health problems ^19^. Findings like these have led to calls for researchers to recognise that universal school-based interventions can lead to negative outcomes in children and young people and to look for different intervention models ^20^.

There are some multicomponent whole-school preventative mental health interventions based on the World Health Organisation ‘Health Promoting Schools’ framework. A recent cluster-RCT evaluated a multicomponent whole-school health promotion intervention delivered in secondary schools by lay counsellors in Bihar, India (SEHER trial). They reported significant improvements in comparison to a control group in measures of school climate, depression, attitudes towards gender equity, bullying and victimisation ^21, 22^. Significant effects on these outcomes were observed at the end of year 1, with further improvements observed after two years. The study further reported that students in intervention schools enjoyed the activities and found them interesting and informative. Previously, an RCT of a similar intervention in Australia (the Gatehouse project) observed a significant reduction in adolescent risk behaviours such as drinking and regular smoking ^23, 24^. We aim to explore the applicability of this ‘Health Promoting Schools’ framework in the UK.

We will work with schools and young people to adapt the SEHER intervention and intervention resources to be appropriate and acceptable to secondary schools in a city in the UK. We will test the adapted intervention and its implementation in a feasibility trial before doing a full trial (aiming to include all secondary schools). A key factor is whether a full trial can be done using mental health data collected as part of the Born in Bradford cohort study (further details to follow).

## Aims and objectives

The study aim is to carry out a feasibility trial to determine if it is possible to implement a cluster-randomised trial of a multi-component whole-school intervention aiming to improve the school climate (PPAMH!). The feasibility trial will not aim to estimate the effect of the intervention, but to learn whether the intervention and research are feasible. A full trial is planned if the intervention is feasible, and the resources are identified.

The specific objectives are to:

1. Establish whether secondary schools can be recruited to take part in the trial.

2. Pilot the randomisation process, including accessing the school-level data for the factors used in randomisation.

3. Assess the acceptability of the PPAMH! intervention to school staff and the well-being mentors involved in implementation using qualitative research methods, including barriers faced and possible solutions.

4. Assess adherence to the PPAMH! intervention by collecting data on the activities undertaken in school (e.g. number of sessions and number attending).

5. Establish whether school absence data and mental health data can be accessed via the education record and the Born in Bradford cohort study respectively.

### Study design

The design is a feasibility cluster-randomised trial of an intervention that aims to create a more positive school environment. The feasibility trial will involve randomisation of six schools with a primary outcome of the successful delivery of the intervention in schools determined by 1) qualitative interview data from staff involved in implementing the intervention and 2) quantitative data on intervention delivery via an activity log including number of sessions and attendees. 

The quantitative outcome data we will access in this study is either routine data from the education record or is data that is already being collected as part of the Born in Bradford cohort study. The cohort study has HRA ethical approval (IRAS number 295413; REC reference number 21/YH/0261) and this approval includes plans for supporting interventions that will use Born in Bradford outcome data. The Born in Bradford study information and informed consent process included information about the use of participant data to evaluate interventions, to ensure that cohort participants are fully informed.

### Research ethics

The study has been reviewed by the Health Sciences’ Research Governance Committee (University of York) and received approval on 15th March 2024 (Ref: HSRGC/2024/616/F). Informed written consent from participating school leaders, school staff and wellbeing mentors will be obtained by the research team. 

### Study outcomes

***Primary outcome***

Successful delivery of the intervention in schools determined by descriptive quantitative data on intervention delivery via an activity log of number of sessions, frequency of sessions, number of attendees and evidence of training to support delivery.

Assessment of qualitative data about implementation via interviews with relevant school staff (and the wellbeing mentors) involved in implementing the intervention.

***Secondary outcomes***

Successful collection of school absences data, as a proxy measure of the school environment. This was selected as the planned primary outcome for the full trial by young people and stakeholders. It will be available for all pupils via the educational record (routine data).

Access to mental health and wellbeing data of year 9 students, collected in the Born in Bradford cohort study, including mental health problems such as depression and anxiety (measured by RCADS-25), mental wellbeing (measured by SWEMWBS) and bullying.

## Methods

### Study setting

The study is located in Bradford, an urban, multicultural city in the North of England, UK. Bradford is one of the largest local authorities in England with a population of 547,000, and has one of the youngest and most diverse populations in the UK ^25^. Within Bradford there are 59 secondary schools, including 7 maintained schools, 31 academies, 11 independent schools, 9 special schools and 1 independent sixth form college.

This study will make use of survey mental health data being collected in Bradford secondary schools as part of the Born in Bradford cohort study ^26^. Born in Bradford’s ‘Age of Wonder’ study is a continuation and expansion of the Born in Bradford longitudinal birth cohort study that began in 2007. Between 2007 and 2011, 12,453 women were recruited who delivered 13,786 live infants ^27^. Half of the Born in Bradford participants live within the fifth most deprived wards for England and Wales, and 45% are of Pakistani origin ^28^. From 2021, Born in Bradford have expanded the cohort to include up to 30,000 young people, with the aim of following these young people through adolescence and into adulthood.

Preliminary results from the Born in Bradford survey of young people in 2022–23 (N ~ 5,000) suggest the prevalence of depression and anxiety in young people is similar to other parts of England, and rates of self-harm and probable eating disorders elevated ^29^. Additionally, school absences and persistent absences (defined as missing 10% or more of school) are higher than regional and national averages, with absence rates continuing to rise despite improvements being seen in other parts of Yorkshire and England ^30^.

### Patient and public involvement

A group of young people (aged 16 to 24), The Bradford Healthy Minds Apprentices were consulted at the design phase of the study in a workshop held in September 2022. This group met again in February 2023 to decide upon the name of the intervention and to design the study logo. In May 2023, the group met to review the implementation details for the intervention including who should deliver the intervention in schools. There will be additional input from young people and school staff to adapt the intervention (details below).

### Eligibility criteria

***Inclusion criteria***

Secondary school in Bradford.

Actively participating in the Born in Bradford cohort study.

***Exclusion criteria***

School is outside of Bradford.

School is not part of the Born in Bradford cohort study.

### Recruitment

Schools will be recruited via existing research links with secondaries in Bradford through the Born in Bradford cohort study ^26^. School staff participants will be recruited once the intervention is underway and the staff relevant to the intervention implementation are identified. Intervention delivery will take place in the schools and will be led by ‘wellbeing mentors’: non-clinical community workers.

### PPAMH! intervention

A school climate intervention was identified as a priority by young people and stakeholders in Bradford. The intervention will be adapted for each school through research involvement activities and workshops with young people and key stakeholders such as school staff. For example, students will be asked to choose the topics of focus in the intervention for each half-term (i.e. six weeks).

The PPAMH! intervention is a multi-level whole school intervention. The intervention’s conceptual framework emphasises the importance of a positive school climate. The name of this adapted intervention ‘Promoting Positive Adolescent Mental Health (PPAMH!) for School Climate’ was chosen by our young person’s advisory group.

A positive school climate can be described as one that features a sense of belonging to the school, a participative school environment, student commitment to academic values, and supportive relationships among school students, teachers, parents, and leadership. Improvements to the school climate are targeted using a multi-level school-based intervention, organised at the whole-school, peer group and individual levels. 

PPAMH! includes four priority areas: promoting social skills; engaging the school community (i.e., pupils, teachers, and parents) in school decision-making processes; improving understanding of health and risk behaviours; and enhancing problem-solving skills. By promoting a positive school climate, supportive relationships and enhancing emotional and mental health knowledge, the intervention aims to improve student mental health and wellbeing (i.e. reduced feelings of depression and less bullying) ^21^. 

The PPAMH! intervention itself will be delivered by two voluntary sector non-clinical community workers (‘well-being mentors’), employed by a local mental health charity and supported by the local NHS Trust. In addition to oversight from the charity, the research team will provide training and regular meetings and site visits for the wellbeing mentors.

A well-being mentor will be in intervention schools 50% of the school week to facilitate whole school activities. They will also run peer group sessions and 1-1 activities with year 9 pupils. Example activities are awareness generation, policy review, workshops and skills training (see Table 1). Each half-term (six weeks) will have a different topic of focus, which will have been selected by the students and school staff. Example topics may include mental health and wellbeing, study skills, and rights and responsibilities.

**Table 1.** Examples of specific activities undertaken to improve school climate in previous trials.

Whole School Level	Peer Group Level	Individual Level
Formation of a School Health Promotion Committee	Elected group meetings to discuss peer concerns, develop plans of action, and organise activities	One-to-one support with problems
Assembly-based skits/ performances	Workshops (eg. study skills for pupils, teacher discipline practices)	Referral to appropriate services
Monthly wall magazine to build knowledge on key issues		
Speak-out box (concerns raised and addressed)		

### Adapting the intervention through PPIE

The intervention needs to be adapted for Bradford, including the intervention components (examples are given in Table 1) and the support that will be provided to schools. We will work with young people and schools to develop an intervention handbook and training package. For every intervention school, we aim to hold three one-hour workshops with secondary school students (10–15 participants per workshop) and three one-hour workshops with relevant school staff (6–8 participants per workshop). These will be in-person at school or online, depending on the preferences of the attendees. Workshop 1 will prioritize the topics to be covered as part of the intervention, such as mental health, relationships and rights and responsibilities. Workshop 2 will seek feedback on the proposed activities that form the intervention. Workshop 3 will discuss the delivery of the intervention and how to engage students, such as assemblies, topic boards and workshops. This information will be used to adapt the intervention handbook and resources. The number of workshops held will depend on the timing of recruitment and randomisation of schools. A pragmatic approach will be taken and fewer workshops may be necessary to fit with the school timetable. 

### Randomisation

Schools will be randomised to either being a control school (three schools) or receiving the intervention (three schools). Schools randomised to the control arm of the pilot trial will not be required to do anything additional. The control condition is ‘usual care’.

Schools will be randomised using minimisation. Minimisation will be undertaken in minimPy (or equivalent package in R) using naïve minimisation. Minimisation factors will include 1) baseline absence rate, 2) number of pupils from non-White ethnic groups and 3) number of female pupils.

The proposed timeline for the study is given in Figure 1. Staff and student workshops with the three intervention schools, where we will adapt the intervention, will take place in the Autumn term 2024 (September through to December).

**Figure 1.**
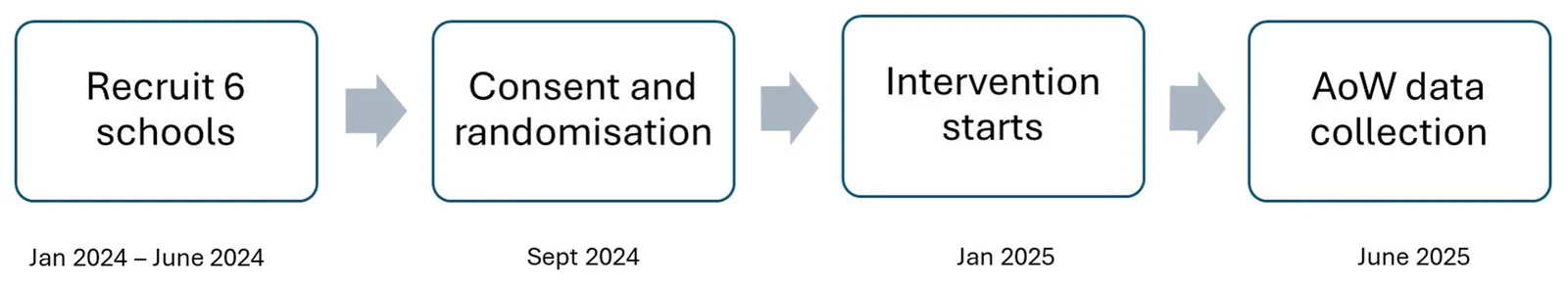
PPAMH! proposed timeline.

### Feasibility of outcome data collection

Should a full trial take place, we will describe characteristics and baseline outcome measures for schools and pupils using routinely collected data (which is generally publicly available via systems such as Get Information about Schools - GOV.UK get-information-schools.service.gov.uk). The primary outcome for the full trial will be school absences taken from the education record (routine data). This will be provided at term and pupil level by participating schools, with minimal additional pupil level variables. We will also collect data on age, year group, sex, ethnicity and eligibility for free school meals. Secondary outcomes include depression and anxiety measured using the Revised Child Anxiety and Depression Scale (RCADS-25) ^31^, wellbeing measured using the Short Warwick-Edinburgh Mental Wellbeing Scale (SWEMWEBS) ^32^ and bullying (bespoke questions). These data are being collected in Bradford schools as part of the Born in Bradford cohort study. For the feasibility trial, we will just determine how feasible it is to access and use this data (i.e. we will not make any comparison in outcomes between intervention and control schools).

### Implementation data collection

Relevant members of school staff who are involved in the intervention implementation will be invited to take part in interviews: one initial long form interview and a series of rapid 15-minute interviews (repeated approximately every six weeks). We would ideally include all staff members involved in implementing the intervention, but at a minimum we would recruit three members of school staff in each school. The ‘wellbeing mentors’ will also be interviewed and in addition will be asked to keep a log of PPAMH! activities undertaken in the school as part of the implementation evaluation. This will include data on the number of sessions, frequency of sessions, number of attendees, evidence of training to support delivery, any adaptations made by the school to the intervention or its delivery, any implementation strategies they have used, and any other relevant information identified by our implementation specialist. 

## Analysis

### Quantitative analysis

The feasibility trial will not include formal statistical analysis. We will describe characteristics of participating schools and the proportion of missing data for the outcomes. We will summarise the quantitative implementation data descriptively. If the results of the feasibility trial show that no major changes are required to the intervention, a full trial is planned. We will publish a protocol for the full trial with detailed analysis plans.

### Qualitative analysis

The initial long-form interview will be transcribed. The rapid response interviews will be audio recorded, listened to, and then summarised into lightning reports. The lightning reports will summarise what is going well, what is not going so well, and any key insights participants have in regard to PPAMH! and its implementation. Lightning reports and long-form interview data will then be coded using the Consolidated Framework for Implementation Research (CFIR) 2.0 and The Expert Recommendations for Implementing Change (ERIC) list. The CFIR analysis will result in a list of barriers and facilitators to implementation. The ERIC will highlight which implementation strategies sites have used to implement PPAMH! and overcome contextual barriers. Adoption and acceptability will also be captured the interview data by asking participants about these directly. Sustainability will be measured in the final rapid interview using the Program Sustainability Assessment Tool, a 15 minute sustainability questionnaire.

Determinants within the CFIR, the implementation strategies listed in ERIC and adoption, and acceptability will be used as codes in NVivo to produce a framework matrix that summarises the information housed under each code at each site. CFIR valence and strength coding will allow us to prioritise barriers and facilitators. We will identify the implementation strategies used to address these. We will then aim to provide a rationale (causal mechanism) for how these strategies worked to address barriers and contribute to our collected implementation outcomes and present this data within implementation research logic models (IRLMs) for each site.

### Progression to a full trial

Data collected on the activities undertaken in schools as part of the intervention, along with the qualitative implementation data, will be used to determine whether a full trial will take place. Each school will have a target for the number of intervention activities completed each half-term/year adapted from the original SEHER trial, for example four awareness-generating assembles per month and two health promotion committee meetings per year ^21^. The intervention activity logs will be compared against these targets. The decision to progress to a full trial will be based on whether these targets have largely been met and if the qualitative analysis of implementation data strongly suggests that school staff and well-being mentors thought the intervention is acceptable and can be implemented in school.

If the quantitative outcome data can be effectively accessed as part of the feasibility trial, then this outcome data will be included in the full trial (i.e. as an internal pilot). If the data access is not feasible or limited due to missing data, then the schools included in the feasibility trial will not be included in the full trial (i.e. an external pilot) ^33^. This will also be the case if the implementation analysis suggests that a substantial amount of modification to the PPAMH! intervention is required for it to be successfully implemented in schools.

## Adverse event reporting

Possible harm as a result of the study will be monitored (e.g. mental distress including self-harm/suicidal behaviour). Standard operating procedures for reporting adverse events will be adapted by the study team. The wellbeing mentors will be prompted to record any adverse events related to the intervention as part of their intervention activity log. The schools’ existing safe-guarding policies will be adhered to. The Trial Steering Committee will review adverse events throughout the trial for safety. If there is evidence of harm due to the intervention or measures used, this may result in a possible recommendation to stop the trial.

## Study limitations

Due to the nature of the intervention it is not possible for schools to be blinded to study allocation. If a full trial is established, the analyst/statistician will be blinded to the intervention status of each school.

Secondary outcomes from the Born in Bradford cohort study are likely to have missing data, principally because of organisational issues in the participating schools on the days of data collection and also due to some pupils being absent on the day of the survey.

## Discussion

This study aims to examine the feasibility of implementing a multi-component whole school intervention for improving the school climate in Bradford secondary schools. The proposed outcome measures of school absence and mental health will be taken from routine data and the Born in Bradford cohort study. This study will explore the feasibility of implementing the intervention and accessing the required data for a full trial. The PPAMH! intervention will be adapted from the SEHER programme trialled in Bihar, India. SEHER has shown promising results in improving the school climate and reducing symptoms of depression ^21– 22^. This is the first time this intervention has been implemented in the UK. The results will inform the decision to move to a full trial and will add to the body of literature evaluating school-based mental health interventions. 

## Ethics and consent

The study has been reviewed by the Health Sciences’ Research Governance Committee (University of York) and received approval on 15th March 2024 (Ref: HSRGC/2024/616/F). Informed written consent from participating school leaders, school staff and wellbeing mentors will be obtained by the research team.

## Data Availability

No data are associated with this article. Figshare: Repository: PPAMH! for School Climate feasibility trial measures, " https://doi.org/10.6084/m9.figshare.27233055 The project contains the following files: The survey questions Figshare: Study materials PPAMH! for school climate feasibility protocol, https://figshare.com/articles/online_resource/Study_materials_PPAMH_for_school_climate_feasibility_protocol/27188265?file=49670706 The project contains the following files: Information sheets Consent form Figshare: SPIRIT Checklist for PPAMH! for School Climate feasibility protocol, https://doi.org/10.6084/m9.figshare.26325616 The project contains the following files: Spirit checklist Data are available under the terms of the Creative Commons Attribution 4.0 International license (CC-BY 4.0)( https://creativecommons.org/licenses/by/4.0/). The Program Sustainability Assessment Tool is available online: https://www.sustaintool.org/psat/assess/
