# Supplementary material for: Promoting Positive Adolescent Mental Health (PPAMH!) for School Climate: protocol for a feasibility randomised control trial in secondary schools
Source: NIHR Open Res. 2026 Jul 23;4:66. Originally published 2024 Oct 24. [Version 2] doi: 10.3310/nihropenres.13678.2 (PMC13710859; doi:10.3310/nihropenres.13678.2)
Supplement: Peer review history

# Reviewer report: Reviewer response for version 2

**DOI:** 10.3310/nihropenres.15706.r43074

**Published:** 2026-10-26

**Authors:** Nicola Byrom

**Affiliations:** 1 King’s College London, London, n, UK, UK

This is important and timely work. With clear government focus on whole-school approaches and school climate as a pathway to improving young people’s mental health, we need robust evidence on what is effective, for whom, and in which contexts. We also need to understand the mechanisms through which change happens. This feasibility trial is a valuable first step towards that evidence.

 My main comment is an encouragement for the team to be more ambitious in how PPAMH! conceptualises a whole-school intervention. The introduction makes a strong case for the Health Promoting Schools framework by positioning it against the limitations of universal school-based mental health interventions: the limited effectiveness of universal CBT-based prevention, and the findings of the MYRIAD trial. The authors will also be well aware of Lucy Foulkes’s work on the potential for mental health awareness-raising activities to have unintended negative effects (e.g. Foulkes & Andrews, 2023).

 The Health Promoting Schools framework is a genuinely structural, settings-based model of health promotion. It treats the school as a setting that continually strengthens its capacity to be a healthy place to live, learn and work. Rather than delivering discrete health programmes, it acts across the whole school: its policies, its physical and social environment, its curriculum and teaching, staff wellbeing, and its partnerships with families and the wider community. In this model, both the problem and the solution lie in the setting. Delivered well, students may benefit without ever being aware that there is an additional spotlight on mental health.

 Reading the description of PPAMH!, I was struck by the emphasis on activities: the number of sessions run and attended, and examples such as assemblies, school magazine features and workshops. These are valuable. On their own, though, they risk resembling the kind of awareness-raising activity the introduction argues against. I was less able to see how the intervention would support schools to examine whether their core operations might be shaping students’ mental health, such as curriculum, pedagogy and assessment. This would require staff to be active participants rather than deliverers. The culture change needed among staff may be as important as, or more important than, any direct activities targetting students.

 It may be that I have underestimated the reach of PPAMH!, and that this structural work is part of the intervention but not fully described here. If so, it would help to make it more visible in the protocol. I would also suggest extending the data collection so that the feasibility trial can assess whether schools are actually making this whole-school shift. This might include, for example:

- reviewing school policies and practices at baseline and follow-up
- staff-reported measures of school climate
- a log of changes to policy or practice made as a result of the programme

Capturing this now would also lay the groundwork for the mechanism questions that a future definitive trial will need to answer.

 Finally, I was curious about the role of students in the process evaluation. As I read it, students are not among those interviewed about implementation. Student participation is a core principle of the Health Promoting Schools framework, and students are best placed to say whether anything about their school feels different. Have the authors considered including student interviews or focus groups? If excluding students was a deliberate decision, it would be helpful to set out the rationale.

Is the study design appropriate for the research question?

Partly

Is the rationale for, and objectives of, the study clearly described?

Yes

Are sufficient details of the methods provided to allow replication by others?

Partly

Are the datasets clearly presented in a useable and accessible format?

Not applicable

Reviewer Expertise:

Public mental health, with a specific focus on emerging adults and the university setting. I was part of the team setting up the University Mental Health Charter, a whole institution framework for improvements in mental health in the university setting.

I confirm that I have read this submission and believe that I have an appropriate level of expertise to confirm that it is of an acceptable scientific standard, however I have significant reservations, as outlined above.

# Reviewer report: Reviewer response for version 2

**DOI:** 10.3310/nihropenres.15706.r41237

**Published:** 2026-07-31

**Authors:** Álfgeir Kristjánsson

**Affiliations:** 1 Department of Social and Behavioral Sciences, School of Public Health, West Virginia University, Morgantown, West Virginia, USA

Thank you for the opportunity to review the revised manuscript. 

 Based on my previous comments and the author’s responses I believe the manuscript is now acceptable for indexing.

Is the study design appropriate for the research question?

Yes

Is the rationale for, and objectives of, the study clearly described?

Yes

Are sufficient details of the methods provided to allow replication by others?

Yes

Are the datasets clearly presented in a useable and accessible format?

Yes

Reviewer Expertise:

Adolescent health promotion, community-based substance use prevention, school health.

I confirm that I have read this submission and believe that I have an appropriate level of expertise to confirm that it is of an acceptable scientific standard.

# Reviewer report: Reviewer response for version 1

**DOI:** 10.3310/nihropenres.14852.r33625

**Published:** 2024-12-17

**Authors:** Katherine Reynolds

**Affiliations:** 1 The University of Melbourne, Melbourne, Victoria, Australia

The manuscript outlines a feasibility trial for a school-wide intervention to improve student mental health and attendance outcomes.  School climate is a central concept but is defined in ways that are not directly aligned to the academic work in this area. For example the definition includes belonging and participation as well as the more typical aspects of academic values and relations; “A positive school climate can be described as one that features a sense of belonging to the school, a participative school environment, student commitment to academic values, and supportive relationships among school students, teachers, parents, and leadership” (p. 6).

 The outcome variables of interest for the project as a whole are

- School absences data
- Born in Bradford cohort study mental health depression and anxiety, mental wellbeing and bullying.

 

 The intervention involves school specific activities and initiatives that relate to four areas

- promoting social skills;
- engaging the school community (i.e., pupils, teachers, and parents) in school decision-making processes;
- improving understanding of health and risk behaviours; and
- enhancing problem-solving skills.

 

 It will involve at least three workshops for student and staff groups ( 6 in total) and other activities co-ordinated by a staff member 0.5FTE whose role is to facilitate whole school activities.

 Many aspects of the feasibility study are clear and well developed.  An aspect that remains less clear is how to assess “school climate” itself in the full trial. The focus is on individual-level student outcomes that are not specific to learning or school belonging or related to the aims of enhancing problem solving and social skills for example. It seems in the full trial additional measures will need to be incorporated to fully investigate the intervention. Also the intervention and activities not being oriented to the school-group -level (e.g., who we are as a group, what do we do here, what do we value) is a missed opportunity.

Is the study design appropriate for the research question?

Partly

Is the rationale for, and objectives of, the study clearly described?

Yes

Are sufficient details of the methods provided to allow replication by others?

Partly

Are the datasets clearly presented in a useable and accessible format?

Partly

Reviewer Expertise:

educational psychology, children and youth well-being and mental health.

I confirm that I have read this submission and believe that I have an appropriate level of expertise to confirm that it is of an acceptable scientific standard, however I have significant reservations, as outlined above.

# Response

**Published:** 2026-07-15

**Authors:** Ruth Wadman

**Affiliations:** Health Sciences, University of York, York, England, UK

The manuscript outlines a feasibility trial for a school-wide intervention to improve student mental health and attendance outcomes.  School climate is a central concept but is defined in ways that are not directly aligned to the academic work in this area. For example the definition includes belonging and participation as well as the more typical aspects of academic values and relations; “A positive school climate can be described as one that features a sense of belonging to the school, a participative school environment, student commitment to academic values, and supportive relationships among school students, teachers, parents, and leadership” (p. 6).

***Thank you for taking the time to review this protocol. Our definition of school climate is taken from the conceptual framework used in the SEHER intervention in Bihar, India. We now reference the Shinde et al 2020 paper.  *** 

 The outcome variables of interest for the project as a whole are

- School absences data
- Born in Bradford cohort study mental health depression and anxiety, mental wellbeing and bullying.

 

 The intervention involves school specific activities and initiatives that relate to four areas

- promoting social skills;
- engaging the school community (i.e., pupils, teachers, and parents) in school decision-making processes;
- improving understanding of health and risk behaviours; and
- enhancing problem-solving skills.

 

 It will involve at least three workshops for student and staff groups ( 6 in total) and other activities co-ordinated by a staff member 0.5FTE whose role is to facilitate whole school activities.

 Many aspects of the feasibility study are clear and well developed.  An aspect that remains less clear is how to assess “school climate” itself in the full trial. The focus is on individual-level student outcomes that are not specific to learning or school belonging or related to the aims of enhancing problem solving and social skills for example. It seems in the full trial additional measures will need to be incorporated to fully investigate the intervention

***Thank you for this feedback. As with the feasibility trial, our aim in the full trial is to utilize routine education data and available cohort data. The available outcome data will depend on the number and geographical spread of the schools included in the full trial. Part of the process in writing the protocol for the full trial will be to identify potential outcome data. For example, the Age of Wonder cohort does not have a specific School Climate measure but has a number of variables aligned to this including school pressure, bullying and whether they enjoy and feel safe school.***

***We have added this to the ‘Progression to a full trial’ subsection:***

***“Part of the protocol development for the full trial will involve identifying appropriate and additional outcome measures within the available data sources. For example, although the Age of Wonder cohort does not include a dedicated measure of school climate, it contains several related indicators, including perceived school pressure, experiences of bullying and sense of safety at school.”***

 Also, the intervention and activities not being oriented to the school-group -level (e.g., who we are as a group, what do we do here, what do we value) is a missed opportunity.

***Thank you for your suggestion. We based the PPAMH! intervention very explicitly on the SEHER intervention trialed in India, including its conceptual framework and intervention activities.  The focus is on school climate, belonging, participation, and relationships. That said, the intervention activities may shape school/year group identity and values. Our qualitative implementation data may be able to identify if this is the case.***

***We have added this as a study limitation:***

***“The intervention comprises individual, group and whole-school activities. It does not include specific activities designed to strengthen shared group identity and values, which may play a role in promoting mental health and wellbeing in school settings. However, this may be captured in the implementation qualitative data.”***

# Reviewer report: Reviewer response for version 1

**DOI:** 10.3310/nihropenres.14852.r33276

**Published:** 2024-11-25

**Authors:** Álfgeir Kristjánsson

**Affiliations:** 1 Department of Social and Behavioral Sciences, School of Public Health, West Virginia University, Morgantown, West Virginia, USA

Thank you for the opportunity to review this manuscript.

 The manuscript describes the feasibility in implementing a pilot study as a preparation for a larger trail. School climate is an important concept that has been shown to relate to mental and health and well being among youth in schools. Although the study report is largely clear and well written, I have a few comments related to levels of impact and analyses that I found challenging.

 Major comments

 - Unclear levels of causality and inference. Apparently the objective of the study is to improve School climate. School Climate is a mezzo level concept and thus requires mezzo level implementation and testing. Its effects are generated from social contextual functions at the school level and should impact whole schools and students. But the measurement of impact and inference in the proposed randomized study are solely based on individual level observations. School level impact appears absent for the study does not include any higher level measurement points and analyses. At a minimum, how school climate is observed solely via individual level observations needs solidified.

 - Challenging argument re individual vs. whole school levels. School Climate appears described with a selection of individual level objectives, such as reducing individual level feelings of depression and bullying delivered via "well-being mentors". How a series of short-term individual level interventions will lead to improved "school climate" at the school level is not clear.

Is the study design appropriate for the research question?

Yes

Is the rationale for, and objectives of, the study clearly described?

Yes

Are sufficient details of the methods provided to allow replication by others?

Yes

Are the datasets clearly presented in a useable and accessible format?

Yes

Reviewer Expertise:

Adolescent health promotion, community-based substance use prevention, school health.

I confirm that I have read this submission and believe that I have an appropriate level of expertise to confirm that it is of an acceptable scientific standard, however I have significant reservations, as outlined above.

# Response

**Published:** 2026-07-15

**Authors:** Ruth Wadman

**Affiliations:** Health Sciences, University of York, York, England, UK

Thank you for the opportunity to review this manuscript.

 The manuscript describes the feasibility in implementing a pilot study as a preparation for a larger trail. School climate is an important concept that has been shown to relate to mental and health and well being among youth in schools. Although the study report is largely clear and well written, I have a few comments related to levels of impact and analyses that I found challenging.

 Major comments

 - Unclear levels of causality and inference. Apparently the objective of the study is to improve School climate. School Climate is a mezzo level concept and thus requires mezzo level implementation and testing. Its effects are generated from social contextual functions at the school level and should impact whole schools and students. But the measurement of impact and inference in the proposed randomized study are solely based on individual level observations. School level impact appears absent for the study does not include any higher level measurement points and analyses. At a minimum, how school climate is observed solely via individual level observations needs solidified.

***Thank you for this comment. We agree that school climate is a school-level concept and that we are looking at individual-level outcome data. There was not a specific measure of school climate included in the Born in Bradford Age of Wonder cohort study. One of our outcome measures is school absence, as a proxy measure of the school environment. This was selected as the planned primary outcome for the full trial by young people and stakeholders. It will be available for all pupils via the educational record (routine data).***

***Part of the process in writing the protocol for the full trial will be to identify potential outcome data. For example, the Age of Wonder cohort has a number of variables aligned to school climate including school pressure, bullying and whether they enjoy and feel safe school.***

***We have added this to the ‘Progression to a full trial’ subsection:***

***“Part of the protocol development for the full trial will involve identifying appropriate and additional outcome measures within the available data sources. For example, although the Age of Wonder cohort does not include a dedicated measure of school climate, it contains several related indicators, including perceived school pressure, experiences of bullying and sense of safety at school.”***

 - Challenging argument re individual vs. whole school levels. School Climate appears described with a selection of individual level objectives, such as reducing individual level feelings of depression and bullying delivered via "well-being mentors". How a series of short-term individual level interventions will lead to improved "school climate" at the school level is not clear.

***Thank you for this comment. Although wellbeing mentors provide support to individual students (signposting and referral), they are embedded within the wider school environment and are expected to influence school climate through multiple intervention activities (see Table 1). We would expect the cumulative effect of these whole school, peer group and individual activities to contribute to a positive school climate, which in turn can reduce levels of depression and bullying. This is following the conceptual model of the SEHER intervention (on which the PPAMH! is based):*** ***https://doi.org/10.1080/16549716.2017.1385284*** ***.***

***We think we have set this out clearly in the ‘PPAMH! intervention’ subsection, but we would be happy to clarify further.***
